# Engineered CARD11–PIK3R3 T‐cell therapies as weapons of cancer mass destruction

**DOI:** 10.1002/mco2.628

**Published:** 2024-07-01

**Authors:** Wanlu Zhang, Min Wu, Yongye Huang

**Affiliations:** ^1^ Key Laboratory of Bioresource Research and Development of Liaoning Province College of Life and Health Sciences Northeastern University Shenyang China; ^2^ Wenzhou Traditional Chinese Medicine Hospital of Zhejiang Chinese Medical University Wenzhou China; ^3^ Wenzhou Institute University of Chinese Academy of Sciences Wenzhou China

## Abstract

Garcia et al. discover a novel immunotherapy approach by engineering naturally occurring mutations in therapeutic T cells to strongly elevate anti‐tumor activity. The authors identify a gene fusion, CARD11–PIK3R3, to increase activator protein 1 and nuclear factor–κB signaling, interleukin‐2 production, and tumor death in vitro and in vivo

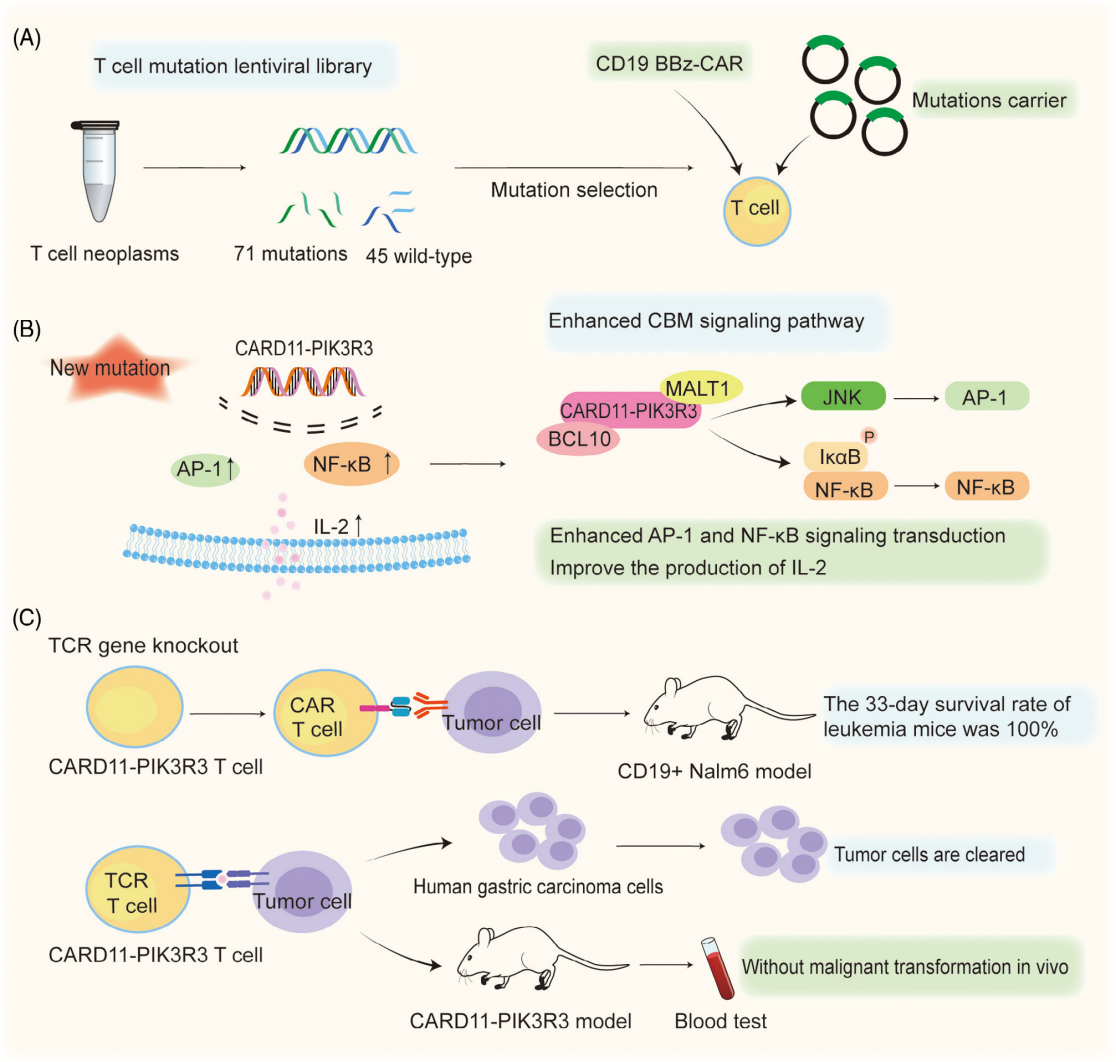

.

1

In a recent study published in *Nature*, Garcia and colleagues describe a novel immunotherapy approach by engineering naturally occurring mutations in therapeutic T cells to enable a strongly elevated anti‐tumor activity.^1^ Specifically, the authors identify a gene fusion, *CARD11–PIK3R3*, which enhances CARD11‐BCL10‐MALT1 (CBM) complex signaling, cytokine production, and anti‐tumor efficacy of therapeutic T cells in immunotherapy‐refractory models.

Immunotherapy, particularly engineered T cells, has ushered in a new era of cancer treatment. As noted by Garcia et al, T‐cell therapy has failed to achieve therapeutic efficacy in 90% of cancers. T cell neoplasms, including T cell lymphomas and clonal T cells in autoinflammatory syndromes, acquire mutations that enhance their fitness and allow for positive selection in an immunosuppressive microenvironment. Many mutations in T‐cell lymphomas have been shown to enhance the potency of T‐cell therapies, thus making use of naturally occurring mutations in T cells for improving T‐cell therapy may be a promising treatment approach. The application of Clustered Regularly Interspaced Short Palindromic Repeats (CRISPR) technology also has great potential for improving the efficiency and safety of engineered T cells in the treatment of patients with refractory cancer. Research has shown that CRISPR‐Cas9 technology can delete the T‐cell receptor (TCR) genes encoding the TCR α chain gene (TRAC) and TCR β gene (TRBC), augment the expression of synthetic cancer‐specific TCR transgene (NY‐ESO‐1), and reduce TCR mismatch.^2^ The study of the characteristics and pathways of T‐cell exhaustion contributes to the success of adoptive T‐cell transfer therapy, and programmed death 1 loss reverses the T‐cell exhaustion phenotype, leading to better anti‐tumor immunity.^2^

Finding new tumor‐associated antigens (TAAs) for engineered T‐cell therapy is an important direction for developing new therapies and overcoming tumor immune evasion mechanisms. Many efforts have been invested in the study of engineered T‐cell therapy in solid tumors. Inducing specific CD8 T cells and exploring specific targets of TCR‐T cells or chimeric antigen receptor (CAR) T cells are important ways to improve the killing efficiency of engineered T cells in cancer. In addition, vaccine administration can induce systemic T cell activation, allowing peripheral CD8 T cells to home to the edge of the tumor; the use of CD3xTRP1 to activate T cells allows them to penetrate deep into the tumor center, thereby playing a key role in tumor suppression.^3^ These findings were recapitulated in several immunotherapy‐refractory tumor models, including CD19^+^ Nalm6 xenograft leukemia model, mesothelioma subcutaneous models (M28), and models expressing human CD19 on B16‐F10 melanoma (B16‐hCD19^+^).^1^ T cells expressing CARD1‐PIK3R3 exhibited superior in vivo therapeutic functions in CAR and TCR transgene‐based models.^1^

The discovery of the role of CARD11‐PIK3R3 in the augmentation of CBM complex signaling in a CD4 cutaneous T cell lymphoma inspired Garcia et al to elucidate how therapeutic T cells exert anti‐tumor efficacy in an antigen‐dependent manner in multiple immunotherapy‐refractory models. Results revealed that CARD11‐PIK3R3 significantly increased activator protein 1 (AP‐1) and nuclear factor–κB (NF‐κB) signaling in T cells, which enhanced the signaling pathways involved in T cell activation and function (Figure 1). Sixty‐one point mutations and 10 gene fusions were cloned into lentiviral constructs for screening to successfully create a T cell mutation library (Figure 1). CARD11‐PIK3R3 mutations enhance the production of interleukin‐2 (IL‐2) in T cells, which drives T cell proliferation and immune responses targeting tumors. Results of the study have demonstrated that the maintenance of memory phenotype and effector function in CD8^+^ T cell subsets with high levels of IL‐2 has been improved, providing advantages for CARD11‐PIK3R3 in screening.^1^ AP‐1 is a dimeric transcription factor from the JUN, FOS, ATF, and MAF protein families.^4^ Based on the cell type and differentiation status, tumor stage, and genetic background of the tumor, AP‐1 exerts oncogenic or anticancer effects by regulating genes associated with cell proliferation, differentiation, apoptosis, angiogenesis, and tumor invasion.^4^

**FIGURE 1:**
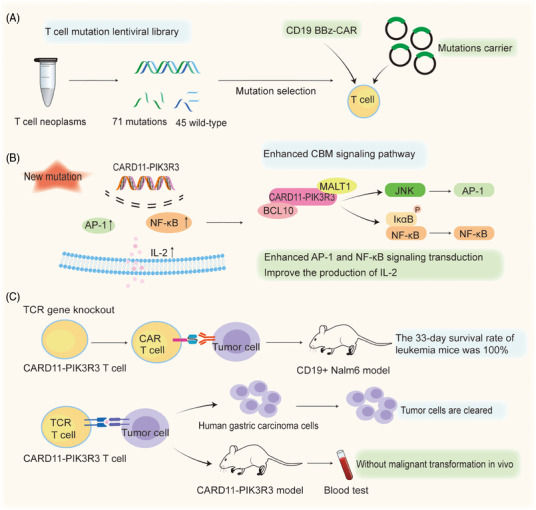
Identification of the CARD11‐PIK3R3 gene fusion and in vivo validation process. (A) Construct T cell mutation lentiviral library screen 71 mutations from T‐cell neoplasms and import the CD19‐BBz‐CAR and mutations carrier into T cells for validation. (B) Fusion of CARD11 and PIK3R3 genes was found to enhance activator protein 1 (AP‐1) and nuclear factor–κB (NF‐κB) signaling by promoting CAM signaling pathways. Meanwhile, the expression of the CARD11‐PIK3R3 mutation can enhance the release level of IL‐2. (C) After T‐cell receptor (TCR) gene knockout, the introduction of CARD11‐PIK3R3 CAR T‐cell therapy cured Nalm6 leukemia in mice with a 100% survival rate at 33 days. The CARD1‐PIK3R3 TCR T cell effectively cleared human gastric tumor cells and controlled tumor cell invasion and metastasis in mice.

The enhancement of CARD11‐PIK3R3 signaling activity augmented the anti‐tumor efficacy of therapeutic CAR‐T and TCR T cells in Garcia and colleagues’ study.^1^ Because the mutation reduces the need for strict lymphatic depletion pretreatment, resulting in the enhancement of T cell anti‐tumor activity and function without extensive pretreatment. CARD11‐PIK3R3 was shown to increase AP‐1 and NF‐κB signaling, IL‐2 production, and tumor death in vitro and in vivo. In layman's terms, enhancing this type of gene fusion alone can strengthen T cells’ tumor‐killing activity without side effects. Moreover, the up‐regulated expression of CARD11‐PIK3R3 in CAR‐T cells promoted the proliferation of the enhanced CAR‐T cells and increased their cytotoxicity, as well as their superior anti‐tumor growth activity. The B16‐F10 melanoma TCR tumor model confirmed that CARD11‐PIK3R3 improves the antitumor efficacy and safety of CAR‐T lymphocytes in an immunosuppressive tumor microenvironment. Furthermore, in a CD19^+^ Nalm6 xenograft leukemia model, treatment with the enhanced CAR‐T cells resulted in 100% survival at 33 days, while treatment with normal CAR‐T cells resulted in a median survival of only 24 days (Figure 1). In TCR T cells, the expression of CARD11‐PIK3R3 also significantly increased the efficiency of T‐cell therapy.

The findings revealed by the CARD11‐PIK3R3 gene fusion mutation in this study are poised to attract huge interest in the field. CARD11‐PIK3R3 holds antigen‐dependent phenotypic effects that not only mitigate the risk of autonomous proliferation but also seem to diminish the necessity for eliminating lymphodepleting chemotherapy. The augmented T‐cell activity selectively targets tumor cells while sparing normal cells, thereby mitigating the detrimental effects of treatment on healthy tissues and consequently reducing the common side effects associated with T‐cell therapy. Simultaneously, the significantly heightened killing capacity of the T cells optimizes the utilization of the patient's own immune system in combating the tumor, thereby substantially enhancing the prospects of successful immunotherapy. Promisingly, in long‐term in vivo xenograft and syngeneic studies across multiple models and at elevated T‐cell doses, CARD11‐PIK3R3 exhibited no signs of lymphoma relapse up to 418 days post‐overdose of T‐cell transplantation. These data unequivocally underscore the advantageous role of the CARD11‐PIK3R3 fusion gene in treating those untreatable cancers.^1^ The amplified killing activity of T cells augments their ability to recognize and eliminate tumor cells, thereby diminishing the likelihood of residual cell survival and reducing the risk of tumor recurrence.

In addition, CARD11‐PIK3R3 T‐cell therapy is likely to be a breakthrough in regard to the bottleneck of conventional T‐cell therapy. Such as, Immune‐related adverse events (irAEs) are common in recipients of engineered T‐cell therapy, which necessitates the use of high‐dose steroids and second‐line immunosuppression. However, the use of immunosuppressive agents can impair the effectiveness of immune checkpoint inhibitors.^5^ Garcia and colleagues focused on the screening and effects of specific mutations like CARD11‐PIK3R3 and their impact on T‐cell signaling and anti‐tumor efficacy, providing clinically applicable novel solutions for improving T‐cell therapy and strategies to avoid irAEs.^1^

In conclusion, this study published in Nature demonstrates that the gene mutation found in lymphoma CARD11‐PIK3R3 increases the killing effect of T cells against solid tumors by a hundredfold. This finding also indicated that rational use of naturally occurring genetic mutations may be a new direction for improved T‐cell immunotherapy. In the future, we need to further explore the potential influence and impact of mutations on T cell phenotype in order to provide a relatively safe and feasible plan for clinical application.

## AUTHOR CONTRIBUTIONS

Y.H. and M.W. provided the conception, funding support, revision, and supervision. W.Z. conducted the literature research, wrote the initial manuscript, and drew the figure. All authors have read and approved to publish the article.

## CONFLICT OF INTEREST STATEMENT

The authors declare no conflict of interest.

## ETHICS STATEMENT

Not applicable.

## Data Availability

Not applicable.
